# Inspection of Underwater Hull Surface Condition Using the Soft Voting Ensemble of the Transfer-Learned Models

**DOI:** 10.3390/s22124392

**Published:** 2022-06-10

**Authors:** Byung Chul Kim, Hoe Chang Kim, Sungho Han, Dong Kyou Park

**Affiliations:** 1 School of Mechanical Engineering, Korea University of Technology and Education, Cheonan 31253, Korea; mir7942@koreatech.ac.kr (B.C.K.); ghlckd12@koreatech.ac.kr (H.C.K.); 2 SLM Global Co., Ltd., Daejeon 34037, Korea; sh.han@slm-global.co.kr; 3 Department of Electromechanical Convergence Engineering, Korea University of Technology and Education, Cheonan 31253, Korea

**Keywords:** hull cleaning condition, underwater inspection image, soft voting ensemble classification, transfer learning

## Abstract

In this study, we propose a method for inspecting the condition of hull surfaces using underwater images acquired from the camera of a remotely controlled underwater vehicle (ROUV). To this end, a soft voting ensemble classifier comprising six well-known convolutional neural network models was used. Using the transfer learning technique, the images of the hull surfaces were used to retrain the six models. The proposed method exhibited an accuracy of 98.13%, a precision of 98.73%, a recall of 97.50%, and an F_1_-score of 98.11% for the classification of the test set. Furthermore, the time taken for the classification of one image was verified to be approximately 56.25 ms, which is applicable to ROUVs that require real-time inspection.

## 1. Introduction

The submerged part of a ship’s hull is susceptible to biofouling in the form of pollutants or organisms such as water mosses and seagrass that attach themselves to the bottom and sides of the submerged surfaces. This phenomenon not only damages the surface of ships but also provides unwanted resistance during normal operation, resulting in inferior performance [1,2,3]. In addition, when a ship enters a port, the various pollutants attached to the hull surface can contaminate the seawater in the port. In the case of ships traveling abroad, aquatic alien creatures that are transported to a different region can disrupt local marine ecosystems [4]. Thus, hull surfaces must be cleaned periodically while ships are anchored in a port.

Conventional hull cleaning is performed by divers. Recently, however, studies have been conducted on cleaning hull surfaces using a remotely operated underwater vehicle (ROUV) [5,6,7,8,9]. A human operator observes the submerged hull surface through a camera mounted on the ROUV and checks its condition. The ROUV is subsequently remotely controlled to clean the affected parts of the hull. However, autonomous hull cleaning without human intervention requires ROUVs capable of recognizing the hull condition. In addition, since the hull condition should be immediately fed back to the ROUV, the process of recognition must occur in real-time.

However, owing to underwater conditions, the images observed by an ROUV through its camera are not clear. In addition, the images may differ depending on the depth of operation, underwater conditions, and lighting; consequently, existing image-processing methods are insufficient for the accurate recognition of hull conditions. Therefore, this study proposes a classification method to recognize the hull condition using convolutional neural networks (CNNs) [10] with images of the hull surface acquired through the ROUV camera. Based on the image, the hull condition is categorized into two classes: (1) positive class: the hull surface is contaminated enough to require cleaning; and (2) negative class: the hull surface is clean enough to not require cleaning.

When using CNN models, both models and training datasets are important. The models used in this study were trained using a publicly available dataset called ImageNet [11]. Therefore, they cannot be used to classify hull surface conditions. Instead, we collected hull surface images under various underwater conditions using our ROUV. However, collecting underwater hull images is difficult because it requires permission from ship owners. In addition, less-than-optimal underwater conditions complicate the process of collecting the images of submerged hull surfaces. Therefore, in this study, we used a transfer learning [12,13] technique to retrain pretrained models for image classification to enable high accuracy with a small number of images. Furthermore, the required training time is short. However, several well-known pretrained models exist for image classification. These can present different classifications for the same image. To overcome this problem and increase accuracy, we used a soft voting ensemble [14] technique comprising transfer-learned models. Finally, to the best of our knowledge, this is the first study on hull surface inspection using machine learning techniques.

The major contributions of this study are as follows:

1. The condition of the hull surface was classified with high accuracy by using CNN models with hull surface images.
2. Our own training dataset was obtained under various underwater conditions using the developed ROUV.
3. Transfer learning of the pretrained models was used to adapt the pretrained models to classification of the hull surface.
4. A higher accuracy was obtained using a soft voting ensemble technique comprising several transfer-learned models.

The remainder of this paper is organized as follows. Section 2 describes previous related studies. Section 3 describes the soft voting ensemble classifier used in this study, and Section 4 describes the generation of the dataset used for training the CNNs. Section 5 discusses the proposed method and experimental results. Finally, Section 6 concludes the paper.

## 2. Related Works

### 2.1. Inspection of Products and Underwater Objects

Several studies have used images from cameras to inspect product defects in various fields. Chang et al. [15] proposed a method for the defect inspection of the color filter, which is a component of the TFT-LCD module, using fuzzy inference from the inspection images. Jiang et al. [16] proposed a method for inspecting printed circuit boards (PCBs) for defects using logistic regression from inspection images. Zhang et al. [17] used a genetic algorithm, artificial neural network, and expert system to inspect copper strip images for defects. Zhao et al. [18] studied the image-based defect inspection of concrete surfaces. Siegel et al. [19] and Mumtaz et al. [20] studied aircraft defect inspection. Amosov et al. [21] studied the defect inspection of rivet joints in aircrafts. Raouf et al. [22] proposed a machine-learning-based fault classification system for the fault detection of rotating vector reducers.

For hull surface inspection, methods using ultrasound [23,24] or sonar [25] have previously been used to inspect coating breakdown, corrosion, and cracks. However, with the improvement in underwater camera performance and image-processing technology, studies on the automatic inspection of hull surfaces have received significant attention. Neghdaripour and Firoozfam [26] proposed a stereo vision system for underwater hull inspections. Navarro et al. [27] proposed a sensor system and a method for detecting defects on a hull surface using thresholds from the images obtained. Fernández-Isla et al. [28] proposed a method for detecting defects from images of a hull surface using wavelet transform. Masi et al. [29] and Ortiz et al. [30] used artificial neural networks to detect corrosion in seabed pipelines and hulls.

Chin et al. [31] classified biofouling images using transfer learning of the Inception V3 model. Gormley et al. [32] classified images of aquatic creatures attached to marine structures using CoralNet [33]. Bloomfield et al. [34] classified images of aquatic creatures using a CNN. Liniger et al. [35] reviewed classification methods using deep learning to categorize marine growth on offshore structures.

In this study, similar to the studies by Chin et al. [31], Gormley et al. [32], and Bloomfield et al. [34], CNNs were used to classify underwater images. However, the classification target in this study was the condition of hull surfaces and not aquatic creatures. This study utilized transfer learning and a soft voting ensemble.

### 2.2. Datasets for Training

Many publicly available datasets exist for training the CNN models. The MNIST dataset [36] contains a set of handwritten digits from zero to nine. It contains 60,000 training images and 10,000 test images. The images are 28 × 28 grayscale images. They are often used to train simple models. ImageNet [11] is the largest image dataset used in computer vision. It contains more than 14 million images and more than 20,000 categories with a typical category, such as “balloon” or “strawberry”. Most image classification studies have used it as a benchmark dataset. The COCO dataset [37] is a large-scale object detection, segmentation, and captioning dataset published by Microsoft. It contains image annotations across 80 categories with over 1.5 million object instances. It is often used as a benchmark algorithm to compare object detection performance. In addition, image datasets for indoor scenes [38], celebrities [39], dog breeds [40], and flowers [41] exist.

A small number of publicly available datasets exist for underwater images. Moreover, these are mainly datasets for aquatic creatures living underwater. Chin et al. [42] shared a dataset with 1326 labeled images divided into 10 classes, such as algae and balanus. Shihavuddin [43] published a dataset for the identification of coral reef species. CoralNet [33] is a dataset used for benthic image analysis. It also functions as a data repository and collaboration platform. This platform for sharing training data can help overcome the lack of available data. O’Bryne et al. [44] presented a method for overcoming the lack of underwater images. They generated a photorealistic synthetic scene of underwater inspection sites using an encoder–decoder model trained with 2500 images.

In this study, images of underwater hull surfaces were required, but there is no publicly available dataset for them. The existing datasets do not consider underwater objects or focus only on aquatic creatures, such as coral reefs. In this study, we collected images using the ROUV by SLM Global [45].

## 3. Architecture for the Classification of Underwater Hull Surface Condition

### 3.1. Problem Definition

The problem to be solved in this study is defined as follows:

*Given a two-dimensional image*x*of hull surfaces that is input through the ROUV’s camera and labeled as clean (negative class) or unclean (positive class), define a binary classifier*$h\left(x\right)$*that can classify the hull condition* via *image* x*,*

where the output of $h\left(x\right)$ indicates the probability $P\left(unclean|x\right)$ that the input image x is unclean. For a given threshold ε, if $P\left(unclean|x\right)>\varepsilon$, x is classified as unclean, and if $P\left(unclean|x\right)\leq \varepsilon$, x is classified as clean.

### 3.2. Soft Voting Ensemble Architecture

**Figure 1:**
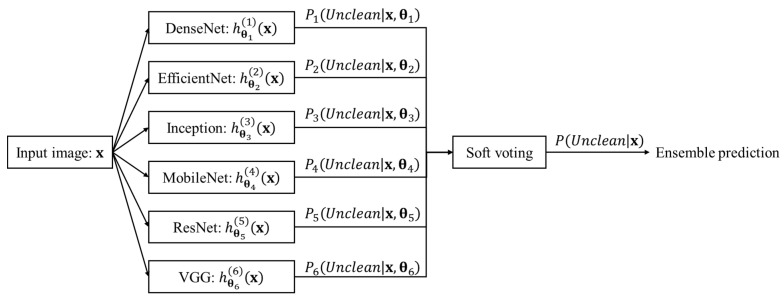
Soft voting ensemble classifier consisting of six models.

In this study, we defined a classifier using a soft voting ensemble of the well-known CNN models DenseNets [46], EfficientNets [47], Inceptions [48,49,50], MobileNets [51,52,53], ResNets [54,55], and VGGs [56], as shown in Figure 1. The soft voting ensemble classifier is a combination of multiple models. In these models, decisions are made by combining individual decisions based on probability values to specify that the data belong to a particular class. [14] In the soft voting ensemble, predictions are weighted based on the classifier’s importance and merged to obtain the sum of weighted probabilities.

The classification method using the soft voting ensemble is as follows:

Step 1. Each of the six models is represented by Equation (1):

(1) $$P_{k}\left(unclean|x,\theta_{k}\right)=h_{\theta_{k}}^{\left(k\right)}\left(x\right),$$

where k is the number representing each model participating in the soft voting, $h_{\theta_{k}}^{\left(k\right)}\left(x\right)$ represents the k-th model for classification, $\theta_{k}$ is the weights of the k-th model, x is the input image, and $P_{k}\left(unclean|x,\theta_{k}\right)\in \left[0,1\right]$—the output value of the k-the model—is the probability that the input image x is clean. In this study, k=1~6 represent DenseNet, EfficientNet, Inception, MobileNet, ResNet, and VGG, respectively.

Step 2. Each model is retrained with our dataset using transfer learning to determine the weights $\theta_{k}$. The dataset creation and transfer learning method are described in Section 3.3 and Section 4, respectively.

Step 3. $P_{k}\left(unclean|x,\theta_{k}\right)$ is evaluated for each model. $P_{k}\left(unclean|x,\theta_{k}\right)$ is the probability that input image x is clean by the k-th classification model.

Step 4. By averaging all $P_{k}\left(unclean|x,\theta_{k}\right)$s, the final prediction value $P\left(unclean|x\right)\in \left[0,1\right]$ is evaluated using Equation (2):

(2) $$P\left(unclean|x\right)=\frac{1}{6}\overset{6}{\underset{k=1}{\sum }}P_{k}\left(unclean|x\right)=\frac{1}{6}\overset{6}{\underset{k=1}{\sum }}h_{\theta_{k}}^{\left(k\right)}\left(x\right).$$

Step 5. Finally, for a given threshold ε, image x is classified as clean if $P\left(unclean|x\right)>\varepsilon$.

Even if the number of models participating in soft voting changes, the overall process does not change. Only the number six in Equation (2) changes to the number of models.

### 3.3. Transfer Learning of the Pretrained Models

The optimal weights of the six models comprising the soft voting ensemble were selected using transfer learning of the pretrained models. Transfer learning [12,13] is a machine learning technique in which a model developed for a task is reused as the starting point for a model for a second task. The six models used in this study comprised 26 sub-models, as shown in Table 1, and they were pretrained for the ImageNet dataset [11]. For each model, optimal hyperparameters and weights were selected through transfer learning and hyperparameter tuning.

Transfer learning is applied as follows. First, the input size of the pre-learned models is redefined to the size of the input image. Subsequently, the pixel values of the input image are normalized to ensure that each pixel value is between 0 and 1. Second, the layers for multiclass classification used in the pretrained models are replaced with layers for binary classification. For this purpose, a global average pooling [57] layer and dropout layer [58] are appended to the last convolution layer of the pretrained model. Finally, a fully connected layer with one node is appended using a sigmoid function as an activation function, as defined in Equation (3):

(3) $$f\left(x\right)=\frac{1}{1+e^{-x}}$$

The redefined models are trained as follows. First, only the weights of the newly appended layers among the layers of the redefined models are tuned by training. Training lasts for 20 epochs with a given learning rate $\alpha_{1}$ for the training dataset, for which the mini-batch gradient descent method is used. The Adam optimizer [59] (with momentum parameters, $\beta_{1}=0.9$, $\beta_{2}=0.999$, $\varepsilon =10^{-7}$) is used as an optimizer. For the loss function, the average of the binary cross-entropy values between the actual label values, $y^{\left(i\right)}$, and predicted values, $h_{\theta_{k}}^{\left(k\right)}\left(x^{\left(i\right)}\right)$, of the images is evaluated, as defined in Equation (4):

(4) $$J\left(\theta \right)=\frac{1}{m}\overset{m}{\underset{i=1}{\sum }}\left[-y^{\left(i\right)}\log\left(h_{\theta_{k}}^{\left(k\right)}\left(x^{\left(i\right)}\right)\right)-\left(1-y^{\left(i\right)}\right)\log\left(1-h_{\theta_{k}}^{\left(k\right)}\left(x^{\left(i\right)}\right)\right)\right],$$

where m is the number of the images used for training.

Following this, the weights of all the layers are fine-tuned for 10 epochs at a new learning rate $\alpha_{2}\left(=\lambda \cdot \alpha_{1}\right)$ that is obtained by reducing the learning rate $\alpha_{1}$ by a factor of $\lambda \left(<1\right)$. Finally, the weights corresponding to the highest validation accuracy among all the epochs are selected.

Optimal hyperparameters such as dropout rate, learning rates, batch size, and sub-models are selected using hyperparameter tuning. First, the hyperparameters are tuned for each sub-model in Table 1. Subsequently, the optimal hyperparameters are selected using a random search method [60]. In this method, the value of each hyperparameter is randomly sampled from the search space comprising them, and the validation accuracy is measured. This is repeated dozens of times for each sub-model. Finally, among the sub-models of each model, the model with the highest validation accuracy was selected.

## 4. Collection and Creation of the Dataset

### 4.1. Description of the ROUV

**Figure 2:**
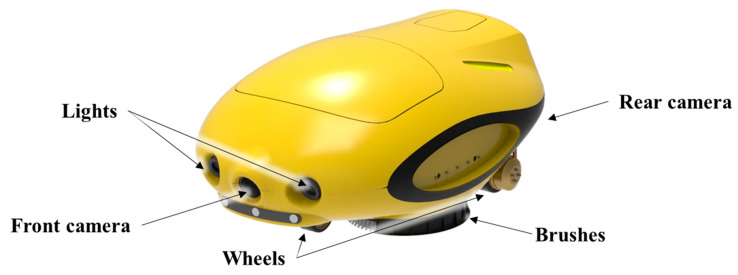
ROUV for underwater hull surface cleaning.

In this study, images of the hull surfaces were collected using the ROUV developed by SLM Global [45] to clean underwater hull surfaces, which is illustrated in Figure 2. The ROUV attaches itself to the hull surface and crawls along it using electrically driven magnetic wheels. It is remotely controlled and monitored by an operator through a tether cable. While moving along the hull surface, the ROUV brushes off the pollutants on the hull surface with two brushes installed at the bottom of the ROUV. The ROUV possesses one camera and two lights in the front and one camera at the rear. The front and rear cameras are used to check the condition of the hull surface before and after cleaning, respectively. The videos are recorded at 10 frames per second (FPS). Table 2 lists the main specifications of the ROUV.

### 4.2. Dataset Creation

To retrain the pretrained models, images of the hull surfaces and their labels are required. The size and number of channels of the images were 512 × 512 and 3, respectively. The labels are represented as either clean or unclean. Images were extracted at intervals of 1 s from the video that was recorded by the ROUV. Subsequently, the images were manually labeled.

**Figure 3:**
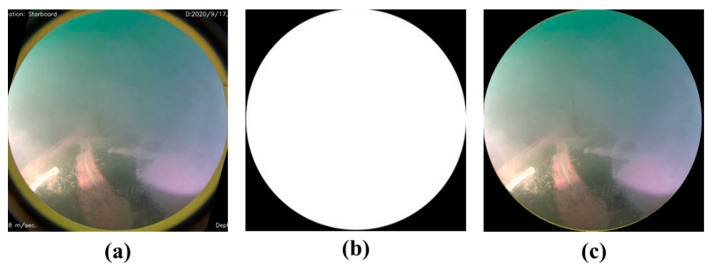
(**a**) Camera image of size 512 × 512, (**b**) mask image, and (**c**) final image used for training.

First, a rectangular area of 512 × 512 pixels containing the camera image was cut from the dashboard image of the ROUV to obtain an image of the camera region, as shown in Figure 3a. As the camera lens has a circular shape, its pure image also has a circular area. To use only the pure camera image, the circular portion is extracted using the Boolean intersection of the front camera image (Figure 3a) and mask (Figure 3b). The resulting image, shown in Figure 3c, was used for training.

**Figure 4:**
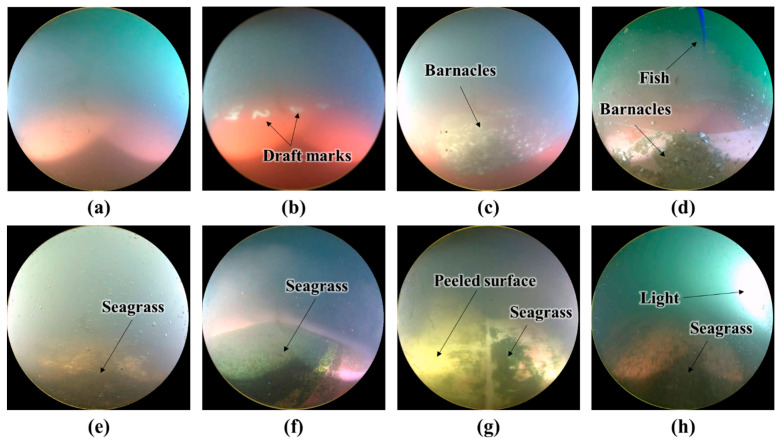
Example images: (**a**,**b**) clean and (**c**–**h**) unclean images.

Figure 4 shows several images from the dataset. Figure 4a,b are clean images, and Figure 4c–h are unclean images. White draft marks are observed in the image in Figure 4b, barnacles are observed in the images in Figure 4c,d, and green mosses are seen in the images in Figure 4e–h. In Figure 4d, the tail fin of a fish can be seen, and in Figure 4g, the coating on the hull surface is shown to have been peeled off. In Figure 4e, the image is obscured by floating matter, and in Figure 4h, the lighting is too strong. As shown in Figure 4, underwater images include various objects, such as draft marks, fish, and floating matter. Furthermore, the underwater conditions such as lighting and the physical state of the hull surface vary. Thus, identifying the hull condition is difficult. Faulty classification due to similarity in colors of different objects is also a concern. For instance, draft marks, peeled sections of the hull surface, and barnacles are all generally white; however, only the images with barnacles should be classified as unclean.

In this study, 5683 images were extracted from videos of 20 hull surfaces at different dates and locations. These were split into two image sets: 2035 clean and 3648 unclean images. To obtain an equal number of images from the two image sets, for training, validation, and testing, 2000 images were randomly selected from each image set. Finally, each image set was split into a training, validation, and testing set in a 60:20:20 ratio. Consequently, for each class, the image set was split into 1200, 400, and 400 images, respectively.

To increase accuracy, the images of the training set were augmented by randomly applying one or more of the following four methods:

1. Brightness adjustment to randomly adjust the brightness of an image;
2. Contrast adjustment to randomly adjust the contrast of an image;
3. Saturation adjustment to randomly adjust the saturation of an image;
4. Cropping to randomly remove a particular region from an image.

**Figure 5:**
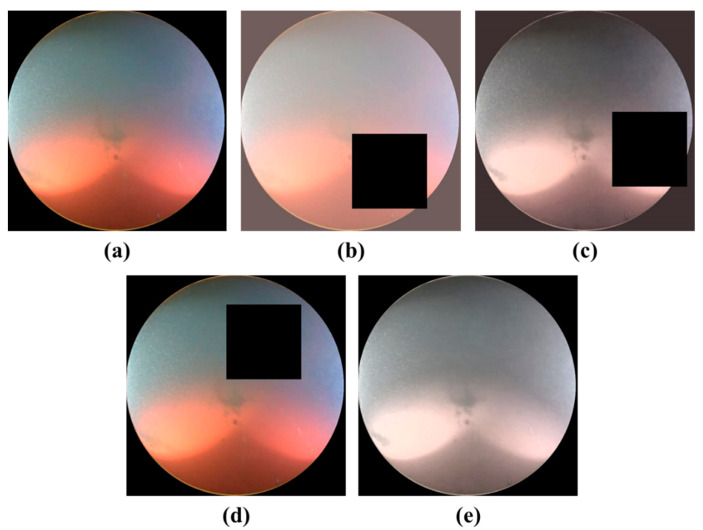
Examples of augmented images: (**a**) original image; (**b**) brightness, contrast, and cropping; (**c**) brightness, contrast, saturation, and cropping; (**d**) cropping; (**e**) saturation.

Considering that the images acquired at the same position on the same hull surface may vary according to the depth and ambient brightness of the seawater, the adjustment of the brightness, contrast, and saturation can improve the accuracy. Cropping can also improve accuracy. However, the commonly used augmentation techniques of translation, rotation, flipping, and scaling were avoided because we experimentally verified that they did not improve the accuracy. We assume that such transformations do not significantly alter the images. Using the aforementioned methods, four augmented images were generated from per image. Figure 5 shows examples of augmented images.

The configuration of the final dataset is listed in Table 3. The test set is used to retrain the pretrained models. The validation set is used to optimize the models via hyperparameter tuning. The test set is used for testing the models and soft voting ensemble classifier.

## 5. Implementation and Experiments

In this study, the proposed soft voting ensemble classifier was implemented using Python and Google’s TensorFlow 2 and was run on computers with an Intel Xeon 3.00 GHz CPU, 128 GB RAM, and two NVIDIA TITAN RTX graphic cards. The pretrained models and weights provided by TensorFlow 2 were used. Retraining and hyperparameter tuning were performed according to the methods described in Section 4. The average time for retraining each model is shown in Table 4.

Table 5 lists the search space for hyperparameter tuning. Some of the values of the batch size in Table 5 may have been selected owing to the memory limitations of the graphic cards.

To determine the optimal values of the hyperparameters for each sub-model in Table 1, 50 samples per sub-model were randomly selected from the values in Table 5. Subsequently, the sub-model was trained with the selected hyperparameter values. For the validation set, the sub-model with the highest accuracy was selected as the optimal model. The accuracy is defined as:

(5) $$Accuracy=\frac{True\;positive+True\;negative}{Total},$$

where the threshold for classification, ε, is set to 0.5. Table 6 lists the optimal hyperparameter values for each model. Table 7 shows that the training and validation accuracies are greater than 98% and 97%, respectively.

Table 8 presents the classification results of the test set using the soft voting ensemble classifier that comprises six optimal models. The precision, recall, and F_1_-score were calculated as:

(6) $$Precision=\frac{True\;positive}{True\;positive+False\;positive},$$

(7) $$Recall=\frac{True\;positive}{True\;positive+False\;negative},\;\mathrm{and}$$

(8) $$F_{1}\;score=2\frac{Precision\times Recall}{Precision+Recall}.$$

Table 8 shows that both the test accuracies and F_1_-scores of the six models are higher than 96%. Therefore, even if used independently for classification, the six models can achieve an accuracy of 96% or higher. The soft voting ensemble classifier has a higher accuracy, precision, and F_1_-score than the six models. Only the recall value of the soft voting ensemble classifier comes behind that of one of the models, i.e., EfficientNet. Therefore, we verified that the images of hull surfaces can be classified with higher accuracy when using the soft voting ensemble classifier.

**Figure 6:**
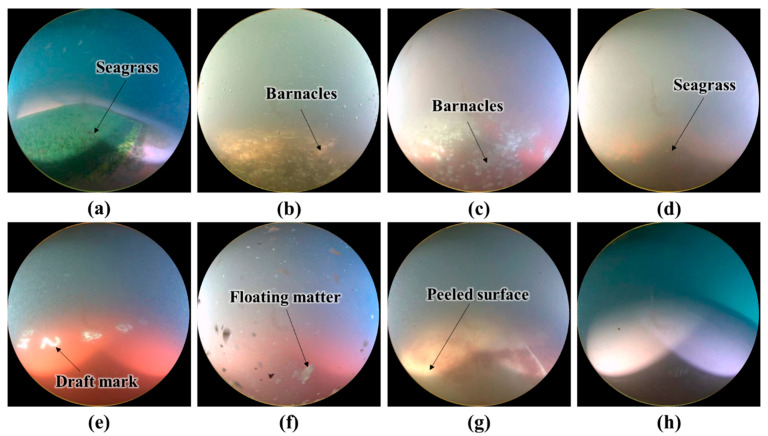
Examples of classified images with the soft voting ensemble: (**a**–**d**) images classified as unclean and (**e**–**h**) images classified as clean.

Figure 6 shows examples of the classification of the images of the underwater hull surfaces using the soft voting ensemble classifier. The soft voting ensemble classifier correctly classifies the images with seagrass and barnacles, shown in Figure 6a–d, as unclean. The images with draft marks, floating matter, and peeled surfaces, shown in Figure 6e–g, were correctly classified as clean. Furthermore, in Figure 6h, the dark surface color due to lighting is not recognized as seagrass but as a clean surface.

**Figure 7:**
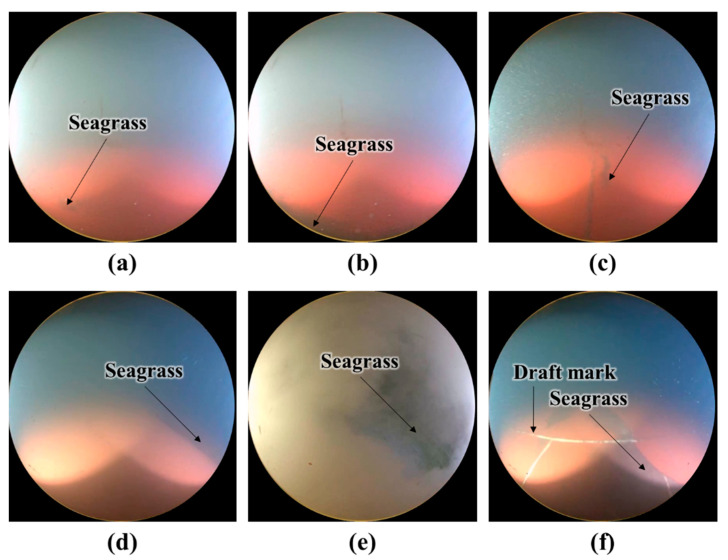
Examples of mis-classified images with the soft voting ensemble classifier: (**a**–**c**) False positive and (**d**–**f**) false negative.

Figure 7 shows examples of the mis-classified images. Since Figure 7a–c only contain a small area of seagrass, the images were labeled as clean. However, the soft voting classifier seems to classify these images as unclean because of the seagrass. In Figure 7d, the dark colored seagrass and seawater overlap; consequently, the hull surface was erroneously identified as seawater. In Figure 7e, the seagrass was not correctly recognized owing to the disturbance caused by the floating matter. The image in Figure 7f has draft marks and seagrass; however, only the seagrass was recognized.

**Figure 8:**
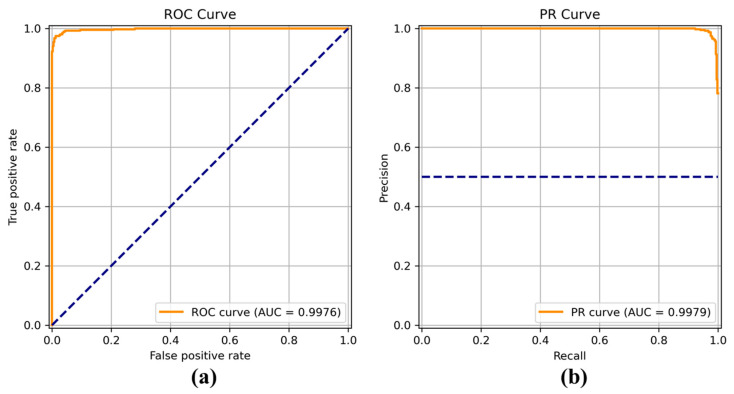
(**a**) Receiver operating characteristic (ROC) curve and (**b**) precision recall (PR) curve of the soft voting ensemble classifier.

**Figure 9:**
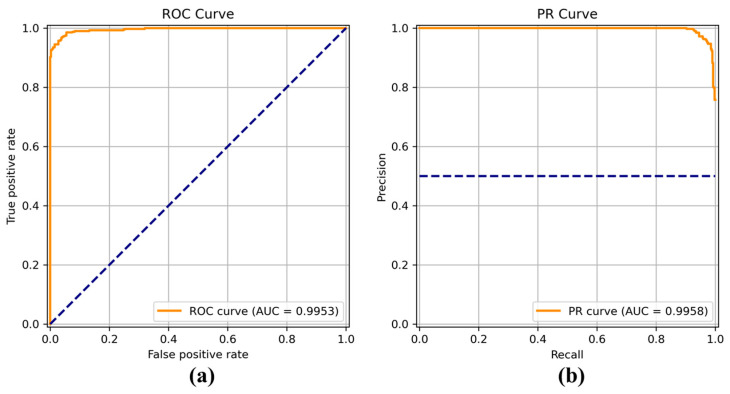
(**a**) Receiver operating characteristic (ROC) curve and (**b**) precision recall (PR) curve of DenseNet.

**Figure 10:**
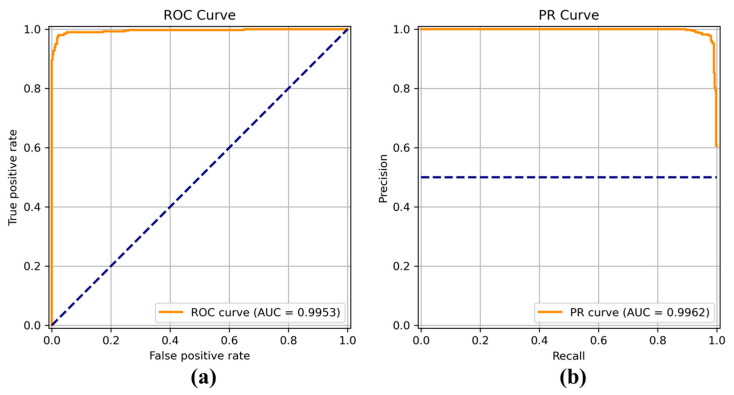
(**a**) Receiver operating characteristic (ROC) curve and (**b**) precision recall (PR) curve of EfficientNet.

**Figure 11:**
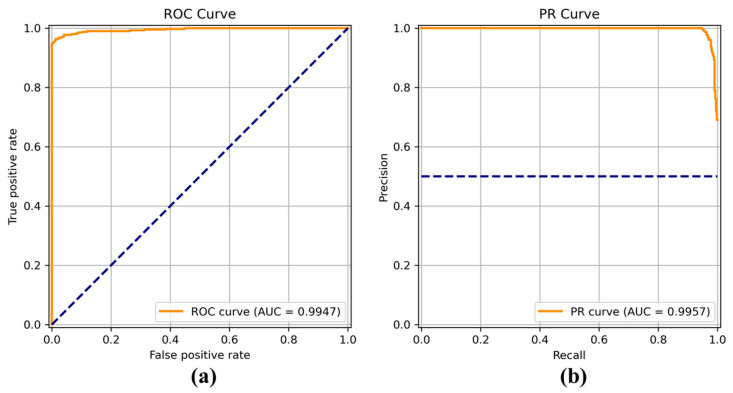
(**a**) Receiver operating characteristic (ROC) curve and (**b**) precision recall (PR) curve of Inception.

**Figure 12:**
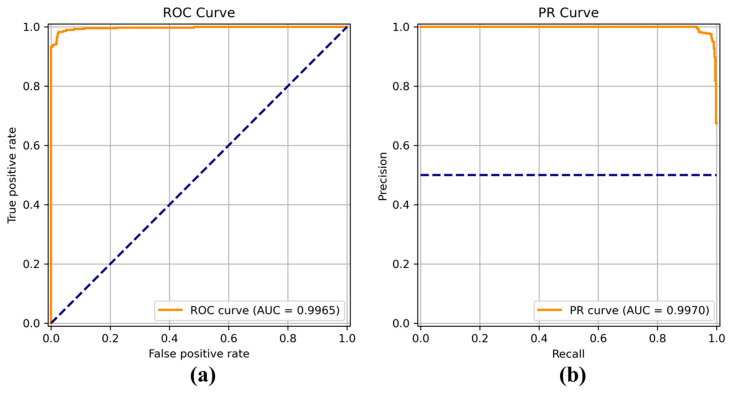
(**a**) Receiver operating characteristic (ROC) curve and (**b**) precision recall (PR) curve of MobileNet.

**Figure 13:**
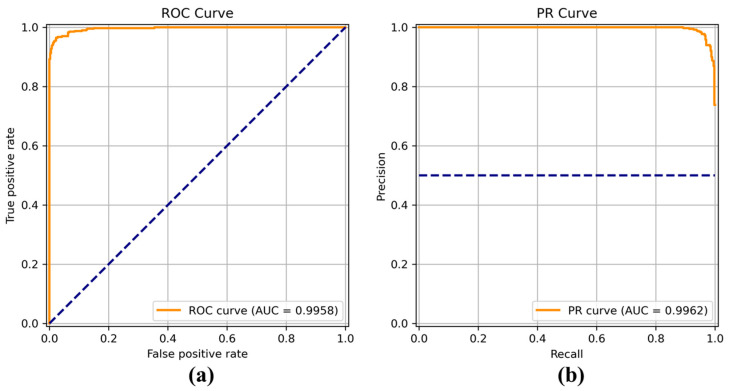
(**a**) Receiver operating characteristic (ROC) curve and (**b**) precision recall (PR) curve of ResNet.

**Figure 14:**
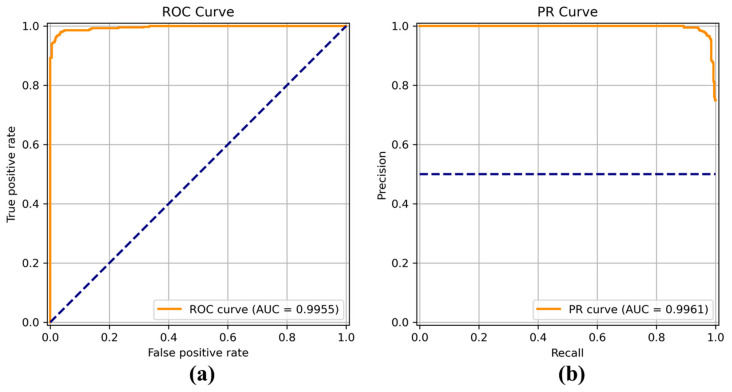
(**a**) Receiver operating characteristic (ROC) curve and (**b**) precision recall (PR) curve of VGG.

Figure 8, Figure 9, Figure 10, Figure 11, Figure 12, Figure 13 and Figure 14 show the receiver operating characteristic (ROC) and precision call (PR) curves for varying classification threshold. For the soft voting ensemble classifier in Figure 8, the area under the curve (AUC) was almost one, and the highest over the other six models. This indicates that the soft voting ensemble classifier has the best ability to classify the conditions of the hull surface among the other six models.

**Figure 15:**
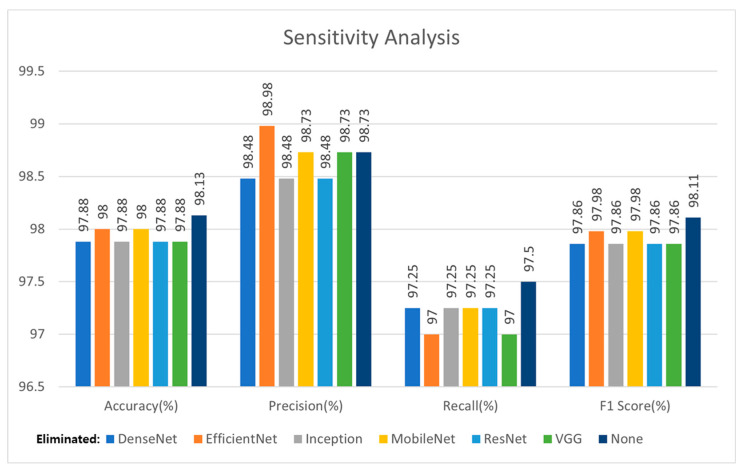
Sensitivity analysis results by eliminating one of the models.

Figure 15 shows the results of the sensitivity analysis, in which one of the models was eliminated. Compared with the results in Table 7, the cases using the five models among the six models were also superior to using only one model. Compared with the case using the six models, except for precision, using the six models (depicted as None in Figure 15) is superior in terms of accuracy, recall, and F_1_-score. The case eliminating EfficientNet is superior in terms of precision but inferior in accuracy, recall, and F_1_-score. Specifically, the F_1_-score, which is the harmonic mean of the precision and recall, of the six models was higher than that of the five models. In conclusion, the case using the six models was superior to that using the five models.

The processing speed for the classification is a key factor for real-time applications. To verify this, the time required to classify an image was measured. Table 9 presents the results of this study. Classifying one image in the test set requires an average of 56.25 ms using only the CPU, which equates to approximately 17 FPS. Based on the speed of the ROUV, images can be sufficiently processed in real-time, even with ROUVs possessing a relatively low CPU performance.

## 6. Conclusions

In this study, a method for inspecting the condition of hull surfaces using images from an ROUV camera was proposed. The classification of images was achieved using a soft voting ensemble classifier comprising six well-known CNN models. To tune the models, they were retrained with images of the hull surfaces. The results of the implementation and experiments showed that the classification accuracy and F_1_-score of the test set were approximately 98.13% and 98.11%, respectively. Furthermore, the proposed method was found to be highly applicable to ROUVs, which require real-time inspection performance.

However, the proposed method requires further improvement. As the dataset used in this study was collected from only a small number of inspection videos, the scope of the results of this study is limited. Therefore, many images that include various types of ships, underwater conditions, and lighting are needed. However, because ship owners are reluctant to provide hull images of their ships, collecting images is difficult. Therefore, in future studies, we plan to apply a data augmentation method using generative models to generate artificial images of the hull surfaces.

## Figures and Tables

**Table 1:** Pretrained models used for the transfer learning.

Model	Sub-Models
DenseNets [46]	DenseNet121, DenseNet169, DenseNet201
EfficientNets [47]	EfficientNetB0~EfficientNetB7
Inceptions	InceptionV3 [48], InceptionResNetV2 [49], Xception [50]
MobileNets	MobileNet [51], MobileNetV2 [52], MobileNetV3-Large [53], MobileNetV3-Small [40]
ResNets	ResNet101 [54], ResNet152 [54], ResNet50 [54], ResNet101V2 [55], ResNet152V2 [55], ResNet50V2 [55]
VGGs [56]	VGG16, VGG19

**Table 2:** Main specifications of the ROUV.

Dimensions	Weight	Power Consumption	Crawling Speed	Cleaning Area Capability
1.5 m × 1.0 m × 0.6 m	270 kg	Less than 15 kW	0~60 cm/s	Maximum 1440 m^2^

**Table 3:** Configuration of the training, validating, and test datasets.

	Training Set	Validation Set	Testing Set	Total
Clean (Negative)	6000	400	400	6800
Unclean (Positive)	6000	400	400	6800
Total	12,000	800	800	13,600

**Table 4:** Average elapsed time for retraining each model.

Models	Average Time for Retraining (Hour)
DenseNet	1.41
EfficientNet	2.75
Inception	0.66
MobileNet	0.58
ResNet	0.69
VGG	0.93

**Table 5:** Search space for the hyperparameter tuning.

Hyperparameters	Values
Sub-models	Sub-models of each model in Table 1
$\mathrm{Initial}\;\mathrm{learning}\;\mathrm{rate}\;(\alpha_{1}$)	0.3, 0.01, 0.03, 0.01, 0.003, 0.001, 0.0003, 0.0001
Learning rate multiplier (δ)	0.1, 0.01, 0.001, 0.0001
Batch size	4, 8, 16, 32, 64, 96, 128

**Table 6:** Optimal hyperparameter values for each model.

**Models**	**Sub-Models**	** $\mathrm{Initial}\;\mathrm{Learning}\;\mathrm{Rate},\;\alpha_{1}$ **	** Learning Rate Multiplier, λ **	**Dropout Rate**	**Batch Size**
DenseNet	DenseNet201	0.003	0.01	0.1	32
EfficientNet	EfficientNetB4	0.01	0.01	0.2	8
Inception	InceptionV3	0.03	0.001	0.1	128
MobileNet	MobileNetV3-Large	0.01	0.01	0.2	32
ResNet	ResNet50V2	0.03	0.001	0.3	32
VGG	VGG16	0.0001	0.1	0.3	16

**Table 7:** Classification results for training and validation sets.

	**DenseNet**	**EfficientNet**	**Inception**	**MobileNet**	**ResNet**	**VGG**
Training accuracy (%)	99.38	98.85	99.58	98.66	99.58	99.45
Validation accuracy (%)	97.88	97.75	97.84	97.00	97.60	97.60

**Table 8:** Classification results using the soft voting ensemble classifier for test set.

	**DenseNet**	**EfficientNet**	**Inception**	**MobileNet**	**ResNet**	**VGG**	**Voting**
True positive	380	392	384	386	382	387	390
False negative	20	8	16	14	18	13	10
False positive	11	9	5	9	8	9	5
True negative	389	391	395	391	392	391	395
Total	800	800	800	800	800	800	800
Accuracy (%)	96.13	97.88	97.38	97.13	96.75	97.25	98.13
Precision (%)	97.19	97.76	98.71	97.72	97.95	97.73	98.73
Recall (%)	95.00	98.00	96.00	96.50	95.50	96.75	97.50
F_1_-score (%)	96.08	97.88	97.34	97.11	96.71	97.24	98.11

**Table 9:** Elapsed time for classifying one image using the soft voting ensemble (CPU only).

	DenseNet	EfficientNet	Inception	MobileNet	ResNet	VGG	Voting
Time (ms)	14.25	14.23	6.90	4.95	6.72	9.20	56.25
FPS	70	70	144	202	148	108	17

## Data Availability

Not applicable.
